# Tumor Organoid and Spheroid Models for Cervical Cancer

**DOI:** 10.3390/cancers15092518

**Published:** 2023-04-27

**Authors:** Ivana Kutle, Robert Polten, Jens Hachenberg, Rüdiger Klapdor, Michael Morgan, Axel Schambach

**Affiliations:** 1 Institute of Experimental Hematology, Hannover Medical School, 30625 Hannover, Germany; kutle.ivana@mh-hannover.de (I.K.); polten.robert@mh-hannover.de (R.P.); hachenberg.jens@mh-hannover.de (J.H.); klapdor.ruediger@mh-hannover.de (R.K.); 2 Department of Obstetrics and Gynecology, Hannover Medical School, 30625 Hannover, Germany; 3 Division of Hematology/Oncology, Boston Children’s Hospital, Harvard Medical School, Boston, MA 02115, USA

**Keywords:** cervical cancer, pre-clinical drug tests, 3D tumor models, tumor spheroids, patient-derived organoids (PDOs), immunotherapy, tumor microenvironment (TME)

## Abstract

**Simple Summary:**

Appropriate testing models are imperative to facilitate the discovery of effective personalized treatments against different cancers, including advanced cervical cancer. This review provides a comprehensive overview of the currently available three-dimensional (3D) models of cervical cancer and their significance in pre-clinical and clinical studies. The review emphasizes the potential of 3D tumor models, such as spheroids from cervical cancer cell lines and patient-derived organoids, to evaluate novel therapies, particularly immunotherapies that target tumor cells and modulate the tumor microenvironment. Notably, the cervical cancer field is underdeveloped regarding use of 3D tumor models, and there is an increasing need to develop appropriate models to address this clinical burden, which will aid in personalized treatment discovery.

**Abstract:**

Cervical cancer is one of the most common malignant diseases in women worldwide. Despite the global introduction of a preventive vaccine against the leading cause of cervical cancer, human papillomavirus (HPV) infection, the incidence of this malignant disease is still very high, especially in economically challenged areas. New advances in cancer therapy, especially the rapid development and application of different immunotherapy strategies, have shown promising pre-clinical and clinical results. However, mortality from advanced stages of cervical cancer remains a significant concern. Precise and thorough evaluation of potential novel anti-cancer therapies in pre-clinical phases is indispensable for efficient development of new, more successful treatment options for cancer patients. Recently, 3D tumor models have become the gold standard in pre-clinical cancer research due to their capacity to better mimic the architecture and microenvironment of tumor tissue as compared to standard two-dimensional (2D) cell cultures. This review will focus on the application of spheroids and patient-derived organoids (PDOs) as tumor models to develop novel therapies against cervical cancer, with an emphasis on the immunotherapies that specifically target cancer cells and modulate the tumor microenvironment (TME).

## 1. Introduction

Cervical cancer represents the fourth most common cancer in women worldwide, with an estimated 604,000 cases and 342,000 deaths in 2020. Currently, one in three-hundred-forty women in Germany dies from cervical cancer. In 2016, 4380 women developed cervical cancer in Germany and there were 1562 deaths. The 5-year and 10-year relative survival rates in 2016 were 67% and 63%, respectively [1].

The distribution of deaths caused by cervical cancer shows an imbalance influenced by wealth, with nine out of ten cervical cancer deaths worldwide occurring in low- and middle-income countries. While this is certainly a reflection of the much better developed cancer prevention system for women in industrialized countries, co-diseases that compromise the immune system also play a crucial role. Women who have HIV, for example, are six times more likely to develop cervical cancer than women who are HIV-negative [2].

Infection with carcinogenic human papillomaviruses (HPV) is crucial for the development of precancerous lesions or cancers. HPV is detected in 99.7% of all cervical cancers and more than 400 different HPV genotypes are now known (PaVE (nih.gov, accessed on 4 April 2023)), of which approximately 40 can infect the genital area (www.hpvcenter.se, accessed on 8 March 2023). Human papillomaviruses are classified according to their risk potential for inducing invasive cervical cancer. A distinction is made between high-risk (HR) and low-risk (LR) types. The four high-risk types, HPV 16, 18, 31, and 45, are associated with approximately 80% of all invasive cancers. HPV 16 alone can be detected in over half of all cancers. The remaining 11 HR types are significantly less likely to be involved in carcinogenesis. LR types cause benign condylomas of the cervix, among others.

Human papillomaviruses belong to the Papillomaviridae family. Mechanisms of squamous cell immortalization and malignant transformation by viral E6 and E7 oncoproteins after HPV infection include inactivation of the tumor suppressors P53 and the retinoblastoma protein (RB), respectively, via proteolytic degradation after binding. For successful infection, HPV must first find a divisible basal or reserve cell of the cervix uteri. As these cells are usually located in the transformation zone, the vast majority of changes occur here. A latent infection of the host cells is often observed, in which the virus infects the cells without causing pathological changes. The oncogenic potency of the virus and the immune status of the host are important for the further course and the possible development of malignancy. In most infected individuals, human papillomavirus appears to clear spontaneously after a period of time without support from therapeutic measures.

Since 1980, both the number of deaths caused by cervical cancers and the incidence of higher tumor stages (≥FIGO stage IIB) have been declining [2]. Depending on the tumor stage, the primary therapy was surgery or radiochemotherapy. In about 12% of cases, there is a primary metastasis at the initial diagnosis. In stage Figo IV, the 10-year survival probability decreases to 16% due to resistance to radiotherapy and chemotherapy, which strongly indicates the need for alternative therapeutic strategies [3,4]. For example, checkpoint inhibitors have become standard treatments in metastatic and recurrent cervical cancer with promising results. However, only a subgroup of patients actually benefits from this therapy. Therefore, additional immunotherapies and new methods to better identify novel immunotherapeutic strategies and estimate potential success are essential to improve patient care. Especially, overcoming barriers that protect tumor cells and create challenges for anti-tumor immune responses, such as the immunosuppressive tumor microenvironment (TME), remain crucial points to be addressed.

## 2. 2D Cancer Models and Importance of TME

In cancer research, 2D cell cultures are most commonly used and often considered to be the golden standard for in vitro studies. To study female reproductive tract malignancies, successful establishment of primary 2D cultures for ovarian [5,6], endometrial [7], fallopian [8], and vaginal [9] cancer have been reported, whereas the cultivation of primary cultures for cervical cancer seems to be challenging, with only a few reports about the successful establishment [10,11] until today. Disadvantages of primary cultures, including limited expansion and short lifespan, prompted the development of cell lines. Based on a recent comprehensive study by Martinez and colleagues, twenty-five cell lines were developed from cervical neoplasias since 1960, of which only nine have identified cervical cancer genomic patterns [12]. However, it has to be considered that, despite the widely accepted standards for regulation, the criteria for the characterization of cell lines derived from cervical neoplasia continue to change. Most in vitro studies on cervical cancer have been completed on HeLa, CaSki, SiHa, or C-33-A cell lines [13].

While convenient, 2D cell cultures fail to recapitulate the complex biological structures that are found within and around tumors, such as interactions with stromal cells and the extracellular matrix, which can also serve as a reservoir for molecules, such as cytokines, lactic acid, miRNAs, and extracellular vesicles (EVs), that are produced by tumors. The combinations of these structures and additional factors such as hypoxia are key to formation of the TME and control (at least partially) how immune cells as well as drugs can access the tumor. Furthermore, characteristics of the TME are thought to have important roles in the inactivation of anti-tumor immune cells and for development of drug resistance. Thus, the TME is not a homogeneous mixture of cancer cells, but rather contains several different types of cells (e.g., cancer stem cells, fibroblasts, mesenchymal stem cells (MSCs), endothelial cells, bone-marrow-derived cells) that contribute to the character of the TME.

The TME has several functions, including orchestration of signals between the tumor and surrounding cells and tissue to maintain the supply of essential nutrients, to promote tumor angiogenesis and possible metastasis, and to protect the tumor from elimination by immune cells by inducing peripheral immune tolerance [14,15,16,17]. One of the major obstacles to development of new therapies with increased effectiveness is the lack of pre-clinical cancer models that properly recapitulate the complexity of human tumors. In this regard, 2D cell cultures have limited ability to mimic the TME in terms of cell morphology, gene expression at the RNA level, and intracellular signaling. Drug resistance between 2D cell cultures and cells in in vivo tumors may differ significantly in this regard [18,19,20].

## 3. Role of 3D Models in Cancer Research

With broadening knowledge about cancer biology and cell biology in general, it became apparent that 2D cell culture systems do not reflect the complexity and heterogeneity of tumors actually present in human disease. Cultivation of cancer cells on plastic dishes under normoxic conditions, constant supply of nutrients, and high passage numbers caused changes in the cell morphology, cytoskeletal organization, cell–cell communication, and accumulation of mutations. Together, these result in modified gene and protein expression patterns that affect cell viability, differentiation, and proliferation processes [21,22,23]. Thus, 2D tumor cell cultures significantly differ from in vivo situations and are often insufficient models for the development of anti-cancer therapies.

Patient-derived xenografts (PDXs) were introduced as a more advanced alternative to model cancer. PDXs are frequently used pre-clinical models of various tumors in which primary cancer cells are transplanted into immunodeficient animals. According to a recent systematic review [24], there are ten studies published with PDX models developed for cervical cancer. In most of these models, cervical cancer tissue from patients was subcutaneously transplanted in severe immunodeficient animals. Such models were useful for the examination of chemotherapy toxicity and efficacy of drug delivery to the tumor site [25,26]. Moreover, models with subrenal capsule transplantation [27,28] and two orthotopic cervical cancer models [29,30] were used to study tumor metastasis and cancer signaling pathways to identify new potential therapeutic targets. Although PDX models offer conserved genetic, histological, and molecular signatures of the original tumor and more closely mimic the TME compared to 2D models, they are still far from the ideal situation, especially considering the lack of infiltrating human immune cells, which are crucial components of the TME. Other important weaknesses include the inability to monitor cellular responses to therapeutic challenges in real time, challenges to predict tumor formation, high costs, high hands-on time requirements, and increased ethical concerns [31].

**Figure 1:**
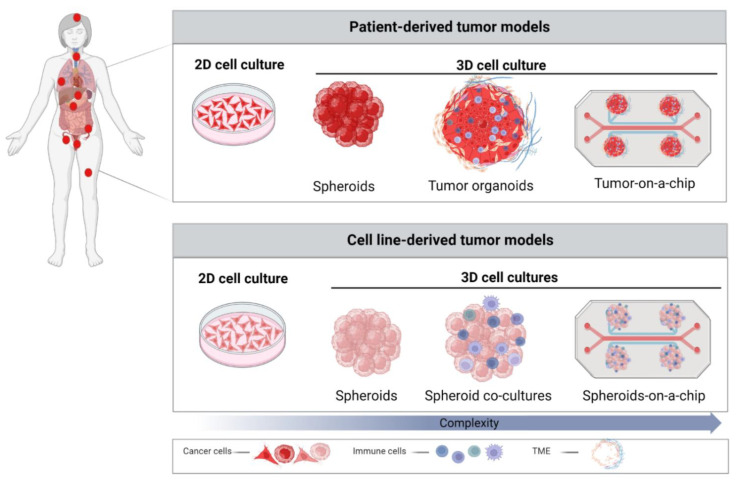
Development of in vitro tumor models for cancer research. Increased complexity of cancer in vitro models from simple 2D cell culture to 3D models, established by introduction of immune cells, extracellular matrix (ECM) proteins, other components of tumor microenvironment (TME), and microfluidic settings as shown. The figure is created with BioRender.com (accessed on 5 April 2023).

These challenges necessitate the introduction of tumor models, which bridge the gap between cell culture and animal models and more accurately reflect in vivo TME. The last decade represented a revolutionary era of 3D cell culture tumor models. Various strategies for 3D models, ranging from spheroids made of cancer cell lines or primary tissue to the recent development of more complex organoid models from tumor explants and microfluidic systems, such as organoids/tumors-on-a-chip, have been developed. Diverse in vitro models applied nowadays in cancer research are depicted based on their complexity in Figure 1.

Spheroids and organoids are the two most commonly used 3D tumor models in pre-clinical cancer research. In this review article, we will focus on these two tumor models and the improvements in their establishment, complexity, and discuss their potential application in the development of therapies against cervical cancer.

### 3.1. Spheroids

Defined as multicellular cellular aggregates that often contain only one cell type, spheroids are the oldest and one of the most commonly used 3D cultures [32,33]. In 1956, Ehrmann and Gey reported cellular aggregates that originated from different cell lines, which were cultivated on collagen isolated from rats [34]. The importance of TME and novel 3D culturing methods was explored during the 1970s and 1980s by several research groups [35,36]. Since the late 2000s, the 3D spheroid culture field has rapidly emerged, which is evident from the drastic increase in the number of techniques developed for spheroid generation. Most methods are relatively simple and can be divided into two basic types. Scaffold-free methods are based on spontaneous cell aggregation, e.g., hanging drop method, agitation-based method in spinner flasks, or seeding the cells on low-attachment surface dishes. The second type of spheroid generation techniques includes facilitated cell aggregation using magnetic levitation, 3D bioprinting, encapsulation in gel-like matrices, and cultivation on natural or synthetic scaffolds. Each method of spheroid generation has advantages and limitations, and the choice of method is largely dependent on the assays in which the spheroids will be applied [37]. For example, the simplest method, such as hanging drop, is very easy to handle; however, it is not the best choice if spheroid size homogeneity plays a crucial role in the assay. While scaffolding-based methods are more complicated to set up, they provide higher uniformity in number and size of spheroids. Multicellular tumor spheroids (MCTS) can be used to model avascular tumors or micro-metastases [38]. In the simplest form, tumor spheroids refer to cell-line-derived 3D cell cultures. With the development of new protocols to isolate cancer cells from solid tumors and advances in the 3D cell culture conditions, primary patient-derived tumor cells are also used for spheroid generation. Incorporation of different cell types into cancer spheroid cultures provides new levels of complexity. It was clearly shown that spheroids can reproduce essential features of solid tumors, such as hypoxia, necrotic core, as well as nutrient (glucose and ATP) and metabolite (lactate) gradients [39]. Therefore, these models can be used to study oxygen impact, diffusion, and exchange of nutrients and other soluble factors, as well as to provide a certain level of heterogeneity of cells within the tumor spheroid [40].

Advances in spheroid production with regard to size control, morphological uniformity, and capacity to integrate multiple cell types or different types of gradients enabled rapid development of high-throughput methods to test new therapeutics with robust reproducibility. Application of tumor spheroids has demonstrated the importance of 3D structures to understand the biology of tumor cells and the mechanisms by which they hijack normal functions of the human body [37]. There are numerous examples of the development of spheroid cultures from cell lines of different types of cancer, such as glioblastoma [41], colorectal cancer [42], lung cancer [43], bone cancer [44], and gynecological cancers, such as breast [45], ovarian [46,47], endometrial [48], and cervical cancer [49]. Diverse generation methods were used to establish spheroids from different cervical cancer cell lines. For instance, HeLa, CaSki, and SiHa spheroid cultures can be easily produced by scaffold-free methods. CaSki and HeLa cells form compact round spheroids, while SiHa cells produce flat and loose spheroids. Further characterization revealed that CaSki spheroids can be used to model aggressive invasion, while SiHa and HeLa spheroids showed slow growth and gradual invasion, respectively [49].

The four main applications of tumor spheroids are (1) cellular functional studies (e.g., cell proliferation, migration, differentiation, and invasion) in an avascular tumor microenvironment [50,51,52], (2) tumor angiogenesis, (3) screening of new anti-tumor therapies, and (4) tumor interactions with immune cells [53,54]. Early studies with spheroids primarily focused on tumor cell biology [38,55] while exploring the potential of spheroid models to mimic tumors in vivo. Independent models showed that gene expression profiles in spheroids more closely resemble in vivo tumor conditions in the context of proliferation, survival, differentiation, and drug resistance [56,57] compared to 2D cell cultures. Migration and invasion assays also revealed crucial similarities of tumor spheroids to in vivo tumor cells and helped to identify major factors in invasion [58,59], which led to development of inhibitors of the metastatic phenotype of tumors, for instance, on the U87 cell line spheroid model for glioblastoma [60].

Tumor angiogenesis is a multi-step process that leads to formation of new blood vessels within tumor tissue. The process allows supply of tumors with oxygen, nutrients, and growth factors and facilitates dissemination to distant organs. Inhibition of angiogenesis is an often-used strategy to prevent the growth of multiple solid tumors by reducing the blood supply to tumor micro-regions, which eventually leads to necrosis within solid tumor tissues. Angiogenesis studies provide an assessment of tumor potential for vascularization, which is often represented by monitoring migration of endothelial cells into spheroids and forming vascular networks within spheroids [61]. Angiogenesis studies typically include tumor spheroids co-cultured with endothelial cells as monolayers or spheroids made from both cell types [61,62,63,64]. Such experimental designs aided understanding of how tumors exploit and rewire angiogenesis mechanisms, which led to discovery of angiogenesis inhibitors as anti-cancer therapeutics [65,66]. Although blocking proangiogenic factors was shown to be an effective strategy to control cervical cancer growth, studies investigating angiogenesis in cervical cancer spheroid models are lacking. It remains to be clearly demonstrated whether results from other cancer types can be transferred to cervical cancer. For example, multiple breast and ovarian cancer spheroid models were developed to study and test angiogenesis and the effects of angiogenesis inhibitors [67,68] and indicated the potential of 3D gynecological cancer models to test and discover new angiogenesis inhibitors. Development of new treatments and drug screening on 3D models in the context of cervical cancer will be further discussed in the following chapters.

Although many features of tumors and complicated tumor microenvironments can be mimicked with cell line- and patient-derived spheroids, the impact of the heterogeneity of tumors, original cell–cell interactions, interactions of tumor cells with the extracellular matrix, and presence of cancer stem cells are necessary for precise pre-clinical (personalized) anti-cancer therapies (Table 1). To overcome those limitations, tumor organoid models were extensively studied.

### 3.2. Patient-Derived Organoids (PDOs)

Organoids are the second most commonly used 3D tumor models generated by the proliferation and self-organization of cells directly isolated from patient tumor tissue. The development of cancer organoids allowed greater retention of the natural cancer cell heterogeneity of the native tumor, thus preserving the pathophysiology of the tumor in vitro, including the genetic and phenotypic features of specific tumor types [69]. In contrast to spheroids, which are formed by facilitated aggregation of cells, tumor organoids spontaneously form 3D structures based on their genetic programming and more closely represent the actual development of tumors. Moreover, organoids are more cost-effective and, in some cases, more physiologically relevant than patient-derived tumor xenograft animal models depending on the experimental design.

The process of organoid generation includes mechanical disruption or chemical digestion of the original tumor tissue. Tumor cell suspensions are then seeded with required growth factors and inhibitors in a culture medium within the extracellular matrix (e.g., matrigel, collagen, or various hydrogels). Different combinations of growth factors and inhibitors in the medium contribute to the generation of distinct lineages in organoids, which was recently described in detail for a breast cancer organoid model [70]. Since the last decade, there have been increasing numbers of successfully cultured tumor organoids originating from various tumor types, including lung [71], prostate [72], breast [73], liver [74], gastric [75], colorectal [76], pancreatic [77], kidney [78], bladder [79], endometrium [80], and several others. The most studied gynecological cancers in the context of organoid models are ovarian and breast cancers. A plethora of different ovarian cancer organoid models based on the histological cancer subtype and cultivation duration were developed [81,82,83]. Breast cancer organoid cultures are even more advanced. Protocols for establishment were extensively explored, which resulted in the generation of numerous organoid cell lines that now serve as organoid biobanks to model disease and test anti-cancer therapies [84,85,86].

In contrast, cervical cancer organoid models remain challenging to establish. There are few reports about their successful establishment and cultivation. In 2020, Maru and colleagues generated organoids of the squamous columnar junction of the normal cervix, but also were the first who established organoid cultures from a rare type of adenocarcinoma, cervix clear cell carcinoma (cCCC). Those organoids reflected histological features, genetic aberrations, and heterogeneity of the original tumor tissue and, thus, represented a promising model to study this rare type of cancer [87]. Another group further advanced the field by establishing long-term organoid cultures from healthy ecto- and endocervical tissue as well as from the associated malignancies from total hysterectomy or Pap smear brush material, respectively [88]. Organoids of healthy tissue represented mini replicates of ecto- and endo-cervical tissue with regard to morphology and transcription profiles and successfully modelled infection with the herpes virus. Cancerous organoids differed in morphology and structure from healthy organoids, recapitulated cancer-associated gene expression patterns, gene alterations, and HPV integration patterns. Recently, Seol and colleagues reported about new cervical cancer organoid cultures. In this study, organoid cultures originated from biopsy materials from four patients with various types of cervical cancer. The histopathological and gene profiles of these organoid models were comparable to the original tumor tissues. Treatment of the cervical organoids with radiotherapy resulted in different responses that reflected the responses observed by the originating tumor types [89]. These three recent achievements in cervical cancer organoid cultures pave the way to the establishment of more organoid cultures and potentially cervical cancer biobanks that could be used to study disease pathogenesis in vitro and to test novel anti-cancer therapeutics in a more personalized approach.

**Table 1:** Advantages and limitations of cervical cancer in vitro models.

Cervical Cancer In Vitro Models	Cell Origin	Application	Advantage	Limitation
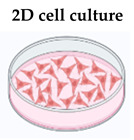	CC cell lines CaSki HeLa SiHa C-33-A	Disease modeling [90] Drug screening [91] Immunotherapy [91] Anti-HPV vaccine [92]	Simple and low-cost maintenance Well-established Simple analysis Long-term cultures Reproducible High-throughput potential	Lack of cell–cell and cell–ECM interaction Lack of natural structures Altered cellular functions Higher sensitivity to drugs Unpredictable for clinical trials
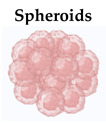	CC cell lines CaSki HeLa SiHa	Disease modeling [52,90,93,94,95] Drug response [96,97,98,99] Immunotherapy [100,101,102] Anti-HPV vaccine [92]	Simple maintenance Moderate costs Cell–cell interaction Preserved cellular functions Mimicking tumor structure Flexible to increase the complexity High-throughput potential	Lack of cell–ECM interactions Lack of heterogeneity Variation in uniformity and reproducibility Challenging analysis
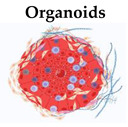	Cancer tissue (SCC, AdCC, neuroendocrine CC)	Disease modeling [88,89,103,104] Personalized therapy [24,89,103] NCT04278326	Natural tumor cell functions Mimicking tumor structure and TME More predictable drug responses High-throughput potential Biobanks establishment Personalized therapy	Difficult to maintain Patient-dependent variation ECM matrix variations High costs Challenging analysis Ethical concern

CC = cervical cancer, SCC = squamous cell cervical carcinoma, AdCC = adenocarcinoma, ECM = extracellular matrix. Graphical elements in table were created with BioRender.com (accessed on 5 April 2023).

## 4. 3D Cervical Cancer Spheroids and Organoids in Translational Research

### 4.1. Drug Discovery

A main goal of academic and industry anti-cancer research is the discovery and development of novel drugs, potentially useful drug combinations, and repurposing of known drugs. Only very few of the drugs shown to successfully combat different types of cancer in pre-clinical development are ultimately translated for clinical use. This necessitates careful evaluation of the selection and screening processes involved already in early stages of drug development. Investigation of the suitability of a drug or drug library to be used against a particular type of cancer typically begins with efficacy studies against well-characterized cell lines established from patients as disease models. Such assays are usually carried out as 2D cell culture models as these are readily amenable to several high-throughput assays, including analysis of proliferation, apoptosis, migration, gene, and protein expression. Lead compounds extracted from such assays are then further evaluated, for example, against primary tissue donated by patients and then in suitable animal models to ascertain initial safety, bioavailability, and efficacy parameters.

Concerning novel compounds screened for their potential benefit in cancer therapy, flavonoids, natural compounds commonly used in traditional Chinese medicine, have shown promising effects against cervical cancer cells [105]. Among these, a study used cervical cancer spheroids to evaluate the anti-cancer effects of the flavonoid naringenin, identifying decreased viability and invasion potential, especially in combination with conventional cisplatin-based chemotherapy [96]. By such combinatorial approaches, future strategies for treating cervical cancer might overcome resistance to conventional chemotherapeutics. Along these lines, expression of long non-coding RNAs (lncRNA) was higher in cervical cancer spheroids compared to parental 2D culture and inhibition of those lncRNAs enhanced cisplatin-induced cytotoxicity [106]. Zhang and colleagues combined imaging strategies with RNA sequencing approaches to improve the prospective treatment of cervical cancer patients [107]. Based on these outcome predictions, the group tested cervical cancer spheroids for their response to multiple treatment options, showing resistance of CaSki spheroids to radiation, but sensitivity to glycolysis inhibition. Apart from cell-line-derived spheroids, patient-derived-organoids offer the possibility to screen for effective therapies before applying them to patients, hence offering personalized treatment options. In one study, organoids were established from patients with cervical cancer and screened for their response to over 170 drugs. Organoid viability was strongly decreased by seven of these compounds, which included proteasome-, histone deacetylase-, and translation inhibitors. The same study used organoids to study their response to chemotherapeutics, and organoids derived from squamous cell carcinoma showed higher sensitivity than those derived from adenocarcinomas and large-cell neuroendocrine carcinomas [107].

Cell–cell interactions, but also interactions between tumor cells and the extracellular matrix ligands, may lead to activation of integrins and other cell adhesion molecules to cause cell-adhesion-mediated drug resistance (CAM-DR) [108,109,110]. Furthermore, 3D printing of the cervical cancer model cell line HeLa in a culture system using gelatin/alginate/fibrinogen hydrogels showed that 3D-printed HeLa cells had higher matrix metalloproteinase expression and greater chemoresistance to paclitaxel as compared to 2D cultures [93]. A 3D bioprinting approach that incorporated adipose decellularized ECM showed that the resulting tumor spheroids had gene and protein expression profiles that more closely resembled in vivo MCF-7 cell line-derived tumors as compared to 2D tumor cell cultures [111]. The in vivo tumorigenicity of the 3D spheroids was also more similar to the original MCF-7 breast cancer cell-line-derived tumors [111]. As further evidence of the contribution of the ECM to greater drug resistance in 3D tumor models, efficiency of docetaxel-loaded PEG-PPMT (poly(ethylene glycol)-poly(ω-pentadecalactone-co-N-methyldiethyleneamine-co-3,3′-thiodipropionate)) nanoparticle (NP) uptake was lower in the 3D tumor model as compared to the 2D cell culture models [111].

Epigenetic regulation is a crucial biological process that occurs during various stages of development and plays a pivotal role in maintaining proper gene expression patterns. During carcinogenesis, aberrant epigenetic reprogramming occurs, which, together with genetic and environmental alterations, promotes tumorigenesis. Aberrant DNA methylation is one of the cancer hallmarks that often results in modified expression of tumor suppressor genes and promotes progression and pathogenesis of cancer [112]. Consequently, epigenetic therapies are regularly applied to treat several different forms of cancer. For instance, epithelial ovarian cancer patients are treated with GSK343, a potent inhibitor of histone methyl transferase EZH2, which successfully overcame platinum therapy resistance. Significant differences in regulation of transcription by specific epigenetic modifiers between 2D and 3D cultures were reported [113]. Accordingly, while treatment of ovarian cancer 2D cell cultures with GSK343 did not result in reduced growth of the cancer cells, treated 3D spheroids of ovarian cancer cells embedded in ECM exhibited diminished cancer cell growth and invasion, as well as increased apoptosis [114]. These data indicate that 3D cancer model systems, as compared to 2D cultures, more accurately resemble in vivo tumors with regard to epigenetic regulation and the influence of epigenetics on drug response [114].

Comparison of EVs derived from 2D and 3D cervical cancer cell culture models showed significantly different EV secretion dynamics and RNA content, with EVs derived from 3D cultures having greater similarity to in vivo circulating EVs obtained from the plasma of cervical cancer patients [94]. This demonstrates the potential of 3D culture systems to mimic the clinical condition and could help provide better models to establish tumor biomarkers and improve our understanding of cervical cancer disease stages.

Further, 3D tumor models based on spheroids are suitable for laboratory testing because of their uniform shape and size. Here, many tests can be performed simultaneously in 96- or 384-well plates, enabling screening of a large number of different therapeutic agents [115]. Cervical cancer spheroid models started to be more often used to assess tumor response and sensitivity to chemotherapeutics [96,116] and drug delivery vehicles [117,118,119]. The drug screening process involves spheroid formation, incubation with a drug, measurement of spheroid integrity and growth kinetics, and cell survival [120]. In this aspect, high-throughput application of tumor spheroids to identify potent drug candidates and reduce animal experiments is highly favorable. Overall, cancer spheroid models showed higher resistance to treatments than 2D culture and recapitulated results frequently observed in solid tumors. Cervical cancer models were conducted on HeLa spheroids and showed higher chemoresistance compared to 2D cervical cancer models [93,98].

Moreover, 3D co-culture models were also used to mimic T cell infiltration of non-small-cell lung cancer tumors [121]. Here, the authors showed changes in the T cell secretome upon tumor infiltration, including proteins related to inflammatory processes caused by cancers, further supporting the importance of intercellular communication in the TME. This 3D model was used to examine the anti-tumor effects of docetaxel, a taxane that causes apoptosis by suppression of microtubule assembly and disassembly, as an initial step towards establishment of a drug screening platform [121]. Moreover, vincristine is a chemotherapeutic that acts by inhibiting tubulin polymerization, with a central role in preventing microtubule formation of mitotic spindles in the metaphase stage. Here, cervical-cancer-derived spheroids have shown decreased sensitivity to vincristine compared to cells cultured in monolayers [97], highlighting the benefits of multidimensional model systems for drug screening.

### 4.2. Pre-Clinical Testing of Immunotherapies

Tumor immune responses and the immune environment play crucial roles in the development and progression of cancer. Major effector cells in anti-cancer immune responses include T- and NK cells, which have been exploited for development of anti-cancer immunotherapies. To better understand tumor immune networks and to test novel immunotherapies, the simplest models containing tumor spheroids co-cultured with immune cells were repeatedly applied for various types of cancers, including ovarian [122], cervical [100], colorectal [123], and lung [124] cancer, where infiltration of immune cells and their cytotoxicity were monitored.

Over the past decades, the FDA approved more than a hundred immunotherapeutic drugs to treat solid cancers. Among those, only three targeted immunotherapies were approved for cervical cancer. Those include a checkpoint inhibitor (pembrolizumab), an angiogenesis inhibitor (bevacizumab), and a tubulin inhibitor in the form of an antibody-drug conjugate (tisotumab vedotin-tftv) (cancer.gov, accessed on 7 March 2023). While immunotherapies, such as checkpoint inhibitors, have shown promising results in multiple solid tumor entities [125,126], the overall response rate remained at only 20 to 40 percent [127]. Concerning cervical cancer, the phase II KEYNOTE-158 study identified an overall objective response rate of only 12 percent for pembrolizumab [128], while the phase I/II Checkmate-358 study showed an objective response rate of 26% for nivolumab [129].

Even before translation into the clinics, the evaluation of immunotherapies commonly shows differences between in vitro and in vivo experiments. While in vitro assays often access cell viability and cytokine release of 2D cell culture models, in vivo studies usually include assessment of more complex murine xenograft models that are often cost-, time-, and labor-intensive [130]. To streamline the development of new and effective immunotherapies for clinical translation, bridging the gap between 2D in vitro and complex in vivo models is essential. In this regard, spheroid and organoid models enable the investigation of immunotherapies in a more complex platform, e.g., by adding organized 3D structures that more closely resemble the respective tumor type and corresponding tumor microenvironment [131].

As an emerging field within immunotherapy, immune cells are equipped with chimeric antigen receptors (CAR) to specifically detect and eliminate target cells. Regarding cervical cancer, a recent study investigated CAR-T cells to redirect the immune cells toward engineered CD19-positive HeLa-derived cervical cancer spheroids, showing target cell lysis in both 2D and 3D conditions [101]. Giannattasio and colleagues [100] used primary NK cells to examine their infiltration into cervical cancer spheroids formed from SiHa or CaSki cells and found that most NK cells surrounded the spheroid, while only a subset of less than 20 percent infiltrated the tumor spheroids. Moreover, spheroid destruction took longer than cytotoxicity experiments performed with the respective 2D cell cultures, potentially due to decreased expression of activating ligands (e.g., NKG2D) on the spheroid surfaces. Another study embedded cervical cancer cells in a 3D collagen hydrogel matrix co-cultured with NK-92 cells to assess cytotoxicity. This experimental design mimicked the physical barrier of solid tumors, leading to reduced migration of NK cells to the cancer cells and decreased cytotoxicity compared to 2D model systems [102]. Due to the strong association of cervical cancer with persistent HPV infections, therapeutic approaches utilizing dendritic cell (DC)-based vaccination strategies were developed, but showed limited clinical success. More complex in vitro systems might facilitate development of such novel strategies. Wang and colleagues [92] compared DC vaccination in 2D and 3D models using an HPV16-E7 DNA vaccine and identified an increase of CD40 and CD80 protein expression, increased levels of IL-12 and IFN-γ, as well as enhanced T cell proliferation in the 3D culture model.

Although several studies reported the establishment of organoids derived from healthy and cancerous cervical tissues [82,87,88,89,103], their utilization for pre-clinical immunotherapeutic studies remains very limited. In contrast, organoids established from ovarian cancer cells were used for immune checkpoint inhibition studies. Here, the authors used a bi-specific antibody targeting PD-1/PD-L1 and observed activation of co-cultured NK and CD8-positive T cells [132]. Moreover, Schnalzger and colleagues used colorectal cancer organoids as a 3D model system to evaluate the cytotoxic effects of CAR-modified NK cells against solid cancer [132]. Patient-derived tumor organoids were also used to study potential combinatorial effects of CAR T cells and birinapant, a second-generation bivalent antagonist of inhibitor of apoptosis proteins (IAP), and showed cancer cell sensitization toward tumor-necrosis-factor-mediated apoptosis [133]. Another study used PDOs to screen and finally enrich tumor-specific T cells. Here, the authors identified T-cell-specific killing of tumor organoids, while the survival of healthy organoids was not affected [133]. Although these studies evaluated organoids for immunotherapeutic treatments of different solid cancer entities, these concepts can be theoretically transferred to cervical cancer.

Multiple clinical studies include patient-derived organoid models to screen for drug responses in vitro prior to treatment. Hence, organoids are used to identify optimal and personalized treatment strategies, e.g., for ovarian [NCT04768270] and cervical [NCT04278326] cancer. Although some clinical studies currently include cancer organoids to test the potential benefit of immunotherapy approaches [NCT03778814, NCT02718235], the use of organoid assessment to direct clinical decisions remains to be approved by regulatory agencies for broader use.

### 4.3. 3D Cultures as a Platform to Test Combinatorial Treatment Strategies against Cervical Cancer

The current therapies for cervical cancer include surgery, radiotherapy, chemotherapy, a few targeted therapies, and several emerging immunotherapy options. However, cervical cancer is a complex disease and current therapies have limited efficacy, which is partially due to tumor drug resistance associated with existing monotherapies [134]. To overcome these challenges, one idea is to combine two or more different therapeutic approaches to achieve improved treatment outcomes. Combinatorial therapeutic strategies may have advantages as they are more likely to inhibit cervical cancer cell survival [134] and reduce duration and adverse effects of high-dose monotherapies. Different combinations of chemotherapy with either radiotherapy, immunotherapy, or targeted therapy are often tested. Most frequently, cervical cancer is treated with a combination of chemo- and radiotherapy [135].

Combining targeted agents with chemotherapy showed increased efficacy against cervical cancer [135,136]. For instance, while VEGF antibody monotherapy showed limited anti-tumor efficacy in cervical cancer in the clinic, combination with standard chemotherapeutic drugs prolonged progression-free and overall survival [135]. The combination of bevacizumab with cisplatin or paclitaxel resulted in more frequent complete response and improved median overall survival of a few months compared to chemotherapy alone [137]. Clinical trials have also investigated the PARP-specific targeted therapeutics veliparib and olaparib in combination with chemotherapeutic agents and showed the feasibility and promising overall response rates in patients with advanced, persistent, or recurrent cervical cancer [138]. Despite these promising results of combining chemotherapy with targeted drugs, results from a number of trials so far have been inconclusive, and, thus, further investigation is required. Positive clinical evidence from combinations of therapeutic approaches used in other cancers supports such initiatives. Effective combination treatments are commonly identified through computational analyses, bioinformatics, and high-throughput screening. Pre-clinical, high-throughput screening of combinatorial therapies with tumor spheroids or PDOs that share similar genetic fingerprints and cellular heterogeneity might be beneficial in identifying the most appropriate therapy in a short time period. In this regard, much more has been completed on ovarian and breast cancer models as compared to cervical cancer. Combination of doxorubicin and immunotherapy with NK cells was tested on various breast-cancer-derived organoid lines where combinatorial effects were evident [139]. Moreover, de Witte and colleagues applied mono- or combinatorial chemo- and targeted therapies on ovarian cancer organoid biobanks and found combinational approaches to be beneficial. Moreover, responses of ovarian organoids were similar to observed responses in patients from which the organoid lines were derived [140]. In the future, such approaches could be applied to cervical cancer organoid biobanks once these are established. In that scenario, potential therapeutic effects could be assessed with a high-throughput assay, as has already been utilized to investigate the cytotoxicity of NK-cell-based immunotherapy on breast cancer 3D spheroid models [141]. A balanced risk–benefit analysis has to be considered to minimize the exposure of patients to severe adverse events due to novel therapies or combinations. Additionally, the costs for such treatments need to be taken into account when considering that many cervical cancer patients live in low-income countries.

## 5. Discussion

As HeLa cells, which originate from the cervical cancer patient tissue, were the first human cell line established, one would expect that cultivation of primary cervical cancer cells and disease modeling would be among the most advanced areas. However, until now, most of the studies in the context of cervical cancer were accomplished on cervical cancer cell lines, with only limited reports of PDX cervical cancer mouse models. There may be several reasons for this. For example, perhaps cervical cancer models are more complicated to establish due to tissue organization. It is also possible that interest is limited as preventative vaccines and very established screening methods are available. In the last ten years, attention to 3D cervical cancer models has been revived. Mostly, the studies were completed on the spheroid models developed from HeLa, SiHa, or CaSki cell lines [49,51] and focused on the biology of tumor cells, invasion potential, and drug response to commonly used chemotherapeutics [96]. However, established as a simple model, 3D spheroid cultures of cervical cancer indicated higher resistance to a particular drug than standard 2D monocultures of the same cell lines [49,93]. Extending the earlier work on organoid culture establishment, recent efforts used whole-exome sequencing and transcriptome analyses to identify upregulated *MYC* expression and activating *KRAS* mutations in patient-derived cervical cancer organoids. Inhibition of oncogenic KRAS signaling by application of the MEK inhibitor trametinib led to apoptosis of the cervical cancer organoids, thus showing the potential of such approaches to direct clinical decisions [142].

**Figure 2:**
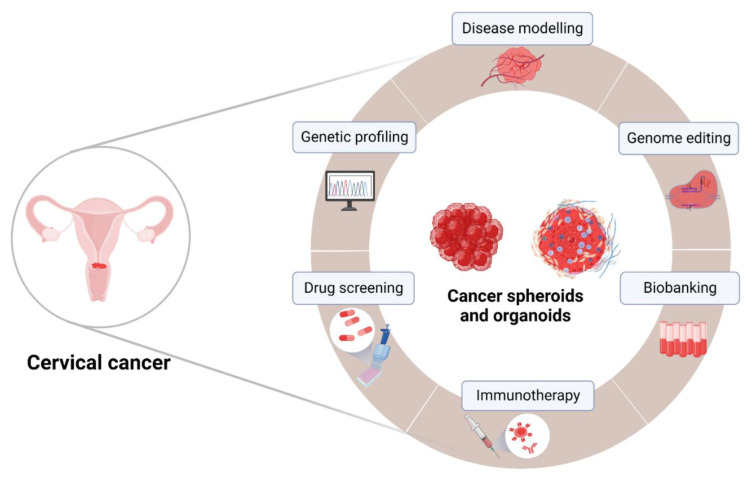
Perspectives for application of 3D models in personalized cervical cancer therapy. Organoids and spheroids originated from cervical cancer samples represent a potential platform to model cervical cancer in vitro, including genetic profiling, genome editing, and storage of patient samples in biobanks as potential open resources and investigation of novel therapeutics. The figure was created with BioRender.com (accessed on 5 April 2023).

Evident changes in the biology of tumor cells cultivated in 2D cell culture conditions and limitations of established PDX animal models, including lack of predictability for clinical trial outcomes and accumulating ethical concerns [143], made 3D spheroid and organoid tumor models highlights of modern oncology. Recent developments in patient-derived spheroids and organoid methods have further improved the reliability of these models in pre-clinical drug screening for cancer patients. Tumor spheroids and organoids enable replication of an in-vivo-like TME within in vitro settings, where the biochemical and physical properties of the TME, including tumor hypoxia, nutrient depletion, acidosis, and heterogeneous gene expression, are remarkably retained [144]. While PDOs are currently utilized in clinical studies to assess personalized treatment options, outcomes of these studies and the potential benefits of organoid models need to be carefully evaluated. Unfortunately, only few clinical studies use organoids in this context, especially in relation to cervical cancer, hence highlighting the need for additional research. Cervical-cancer-derived organoids and spheroids can be genetically profiled and used in pre-clinical and clinical studies, including development of more personalized therapeutic approaches (Figure 2).

Although they offer many advantages in disease modeling and for testing new therapeutics, spheroid and organoid cancer models require higher consumption of reagents and time than 2D cultures. Observed variability between experiments depends on patient-derived materials, the origin of the extracellular matrix [133], and the absence of tumor stroma and vasculature. Patient-derived 3D cultures often contain altered cellular compositions as a result of surgery, mechanical signals, for example, due to shear stress or pressure, as well as other environmental influences [145]. These limitations can result in variations in growth and response to therapy, making it challenging to reproduce the same results across different experiments. Therefore, careful consideration is also necessary when interpreting data obtained from patient-derived material.

To achieve the full potential of 3D cancer models, new methods and techniques provided novel ways to generate 3D microtumors using well-defined xeno-free matrices and facilitate their potential clinical applications. Additionally, manufacturing techniques for tumor spheroids aim to increase uniformity and reproducibility because high-throughput drug screening platforms with heterogeneous tumor spheroids have demonstrated that variability in spheroid size and density significantly affects assay results.

Additionally, there is still a significant need for standardized tests for spheroid imaging, analysis, quantification, and automation for drug screening. The most commonly used fluorescence microscopy method, confocal microscopy, has a small penetration depth that prevents the visualization of large tumor spheroids [146]. Recent advances in non-invasive automated imaging methods and integration of artificial intelligence greatly accelerated analysis of spheroid and organoid assays.

To help avoid bias, errors, and achieve faster analysis of 3D tumor models, essential computer-aided analyses are becoming more widely available. Several artificial intelligence (AI)-based recognition techniques have been developed that incorporate integrated algorithms, including auto-recognition, auto-focusing, and various parameters to assess morphological components, as well as behavioral parameters, such as invasiveness, of organoid and spheroid tumor models [147,148].

Intrinsic limitations of cancer spheroid and organoid systems also include the lack of supporting stroma and blood vessels. Tumor organoids-on-chips and co-cultures with stromal cells were developed to solve the lack of tumor vascularization and stroma. Optimally, the TME within patient-derived spheroids and organoids would contain the entire diversity of immune suppressive cells, including tumor-associated macrophages, myeloid-derived suppressor cells, regulatory T cells, tumor-associated DCs, and other innate immune cells, and might conceivably incorporate tumor-infiltrating immune cell populations.

Two highly important aspects of tumor progression, circulating tumor cells and metastasis, remain experimentally challenging to model in vitro. In 2015, a 4D lung cancer model was developed by introducing the flow of circulating cancer cells as a fourth dimension, resulting in the successful modelling of tumor growth and metastasis [149]. Progression of most tumors can be influenced by different environmental factors, such as diet, exercise, time, microbiome, and altitude. In this direction, there are already 4D spheroid models established for gastric cancer [150] and a glioblastoma organoid model [151] that consider stress stimuli and cell cycle status, respectively. Further advancement on these models may lead to a greater comprehension of tumor biology and, in turn, enable the development of more efficient therapeutic interventions.

## 6. Conclusions

The ability to model the TME in vitro using tumor spheroids will greatly benefit therapeutic development, such as in vitro screening and optimization of pharmacological therapies. This will also provide a platform to elucidate the molecular pathways associated with solid tumor malignancy. Although some limitations remain, recent advances in 3D bioprinting, tumor-on-a-chip, and other microfabrication technologies will accelerate the development of more biologically relevant in vitro TME models. As discussed earlier, animal models have certain limitations in mimicking the highly complex process of human carcinogenesis, physiology, and progression. Furthermore, based on the recent FDA decision, animal models are no longer required to validate all drugs before human trials [152]. Ultimately, cancer organoids are becoming the new gold standard in vitro model with high translational potential. As shown in Figure 2, cervical cancer spheroids and organoids may provide the tools to improve our understanding of the complex interactions relevant to tumor biology and increase the success rate of clinical translation of potential anti-cancer strategies discovered in in vitro experiments. We look forward to a world in which “off-the-shelf” immunotherapies will be economically feasible for broad distribution to patients who desperately need them. Current worldwide efforts to develop and deliver these therapies will hopefully address the medical needs of cervical cancer patients.
